# Antifungal strains and gene mapping of secondary metabolites in mangrove sediments from Semarang city and Karimunjawa islands, Indonesia

**DOI:** 10.3934/microbiol.2021030

**Published:** 2021-12-08

**Authors:** Delianis Pringgenies, Wilis Ari Setyati

**Affiliations:** Department of Marine Science, Faculty of Fisheries and Marine Sciences, Universitas Diponegoro, Semarang, 50275, Indonesia.

**Keywords:** antifungal strains, mangrove, *Pseudomonas aeruginosa*, sediment, *Zhouia amylolytica*

## Abstract

Infection caused by pathogenic fungal species is one of the most challenging disease to be tackled today. The antifungal bacteria candidate can be found in terrestrial as well as aquatic ecosystems, with mangrove forests being one of them. The purpose of this study is to obtain candidate isolates of antifungal strains with a detection approach and gene mapping simulation of bioactive compounds producers and screening to determine qualitative antifungal activity. The research will be carried out by collecting sediment samples from the mangrove ecosystems of Karimunjawa and Mangkang sub-district of Semarang city, isolating and purifying bacteria with Humic Acid Vitamin Agar (HVA), International Streptomyces Project 1 (ISP 1) and Zobell (Marine Agar). added with antibiotics, qualitative antifungal ability screening of each isolate obtained, detection of the presence of PKS gene and NRPS using special primers using the Polymerase Chain Reaction (PCR) method, and molecular identification of each isolate by 16s rRNA sequencing method. Of the total 59 isolates produced from the sample isolation process, 31 isolates from Karimunjawa sediments and 8 isolates from Semarang sediments showed activity against test pathogenic bacteria, namely *Candida albicans*, *Trichoderma* sp., and *Aspergillus niger*. Detection of Biosynthethic Gene Cluster (BGC) showed that the genes encoding secondary metabolites (NRPS, PKS 1 and PKS 2) were detected in KI 2-2 isolates from Karimunjawa. NRPS were detected only in isolates SP 3-9, SH 3-4, KI 1-6, KI 2-2, KI 2-4. The secondary metabolite-encoding gene, PKS1, was detected in isolates SP 3-5, SP 3-8, KI 2-2. PKS II genes were detected only on isolates SP 2-4, SH 3-8, KI 1-6, KI 2-2, and KI 2-4. Isolate SP 3-5 was revealed as *Pseudomonas aeruginosa* (93.14%), isolate SP 2-4 was *Zhouia amylolytica* strain HN-181 (100%) and isolate SP 3-8 was *P. aeruginosa* strain QK -2 (100%).

## 1. Introduction

Mangroves have many marine organisms [1] and have an element content sources [2] and amino acid contents [3], antimicrobial potency [4], tannins and phosphorus [5] and antifungal secondary metabolites [6]. Infection caused by pathogenic fungal species is one of the most challenging disease to be tackled today. The increase in pathogenic fungi-induced infections are worsen with the rise in demand for antifungal substances which may cause harmful side effects, are no longer effective against new pathogenic strains, or even accelerate the rate of resistance to treatment [7]. Antifungal strains produce compounds that show promise in their application as treatments for infections caused by fungi, viruses, and bacteria as well as drugs in the therapy of various types of cancer. Bioactive compounds are generally a product of secondary metabolic processes, from which the term secondary metabolite is derived. Antibacterial compounds can be found naturally on land and water, one of which is in mangrove sediments [8].

The challenges that are often faced by researchers in an effort to obtain new drug alternatives are the rediscovery of known compounds and the significant investment of time and money [9]. Rapid developments in the fields of genomics, bioinformatics, metabolic engineering, and synthetic biology have opened up opportunities for the discovery of new alternative compounds that can be applied as antibiotics or as new types of drugs [10]. One of the methods used to search for candidate antifungal strains that have the potential to produce bioactive compounds is screening based on the presence and mapping of PKS (Polyketide Synthetase) and NRPS (Nonribosomal Peptide Synthetase) genes.

This study aims to obtain candidate isolates of bacterial strains with antifungal activity against pathogenic strains using a detection and simulation approach to map bioactive compounds-producing genes and screening for qualitative antifungal activity. The innovations and advantages in this research are: (1) Antibacterial strains isolated from mangrove sediments are known to produce good bioactive compounds; (2) Activation of silent genes uses several optimization experiments to produce new compounds with antifungal activity.

## 2. Materials and methods

### 2.1. Site and time research

The sediment samples were collected in May to July 2021 from two sites, namely Karimunjawa National Park, Jepara regency and Mangkang sub-district, Semarang city, Central Java. The reason for the sample collection in 2 different places is an area without factory pollution (Karimunjawa islands) and an area with many factories.

### 2.2. Procedure of research

#### 2.2.1. Isolation of antifungal strains from mangrove sediment samples

The treatment of sediment samples until bacterial isolation was carried out with methodologies which refers to Davies-Bolorunduro et al. [11] with several modifications. Bacterial isolation was carried out using the spread plate method with multi stage dilutions by diluting 1 g of sediment sample in 9 mL of sterile seawater. Afterwards, this dilution was repeated by adding 1 mL of the first dilution into 9 mL of sterile seawater, and repeated up to three iterations. 50 µL of the dilution was taken and spread on the surface of three different media types, namely Peptone Yeast Agar (PYA), International Streptomyces Project 1 (ISP 1) [12], and Humic Acid Vitamin Agar (HVA) [13]. The antibiotics Nystatin (2.5 mg/L) and Nalidixic acid (60 mg/L) were introduced to the media in the preparation process. The results of the isolation were incubated at a temperature of 29–34 °C for 7–14 days.

#### 2.2.2. Characterization and purification of bacteria

Bacterial characterization was carried out on the isolated samples. Purification of the bacterial isolates was then carried out according to the method described in Davies-Bolorunduro et al. [11], with several modifications. Bacterial samples that have been cultured on isolation media are grouped according to their morphology. Each isolate was cultured on new media by streak plate method, which was then re-incubated at a temperature of 29–34 °C for 1–5 weeks.

#### 2.2.3. Antifungal activity screening

Screening of bacterial samples with antifungal activity was carried out using the agar plug method based on the work with several modifications [14]. The pathogenic species tested consisted of *Candida albicans*, *Trichoderma* sp., and *Aspergillus niger* which were collected from the Laboratory of the Faculty of Medicine, Universitas Diponegoro, Semarang. The bacterial sample culture that had been incubated for 2 weeks was cut into a cylindrical shape (about 7 mm in diameter), then attached to the surface of Potato Dextrose Agar (PDA) media that had been inoculated with the test pathogenic fungus. The test samples were incubated at a temperature of 29–34 °C Observations and measurements of the inhibition zone were carried out after 72 h of incubation.

#### 2.2.4. Extraction of antibacterial strain DNA from the samples

The Chelex method was used in the extraction of antibacterial strain DNA from the samples [15]. 2–3 inoculation loop of bacterial colonies were taken and introduced in a mixture of 500 µL of 0.5% saponin solution (in Phosphate Buffer Saline) and 100 µL of ddH_2_O. Samples were soaked for 12–24 hours at 4 °C to lyse the cell walls of actinomycetes.

Samples that have been soaked using saponins were processed using a centrifugal machine at 9000 rpm for 15 minutes. The supernatant from the centrifuge was discarded, then 1 mL of PBS solution was added to the natant, then the mixture was vortexed until homogeneous. The homogeneous mixture of natant and PBS was then put back in the centrifugal machine for 10 minutes. The resulting supernatant was removed. 100 µL of ddH_2_O and 50 µL of Chelex 20% solution (vortex Chelex solution before use) were added to the remaining natant. The samples were then heated at 95 °C for 5 minutes, then vortexed, after which they were reheated at 95 °C for 5 minutes. The samples were re-centrifuged for 15 min, then the supernatant was transferred to fresh 1.5 mL microtubes and were ready to be used as a DNA template.

#### 2.2.5. NRPS gene amplification

Detection of the NRPS gene by PCR method was achieved using primer pairs DKF (5′-AAGGCNGGCGSBGCSTAYSTGCC-3′) and MTR (5′-TTGGGBIKBCCGGTSGINCCSGAGGTG-3′) [15], 10 mM each, 0.5 L, which was mixed with 7.5 L Thermo Scientific2X Phire Plant Direct PCR Master Mix, 6 l ddH_2_O, and 0.5 L DNA template of each isolate. The amplification process was carried out in 40 cycles, as follows: initial denaturation stage (95 °C, 5 min), followed by 10 cycles of denaturation stages (95 °C, 1 min), annealing (60 °C, 30 sec), extension stage (72 °C, 1 min), then 30 cycles of denaturation (95 °C, 1 min), annealing (40 °C, 30 sec), extension stage (72 °C, 1 min), and final extension (72 °C, 10 min).

#### 2.2.6. PKS-I and PKS-II gene amplification

Detection of the PKS-I gene was carried out by amplification of DNA Template as much as 1 l with primers MDPQQR f (5′-RTRGAYCCNCAGCAICG-3′) and HGTGT r (5′-VGTNCCNGTGCCRTG-3′) (El Samak et al., 2018), with concentrations 10 mM, 0.5 µL each, 7.5 µL Thermo Scientific2X Phire Plant Direct PCR Master Mix, and 6 µL ddH2O, and 0.5 µL DNA template from each actinomycetes isolate. The amplification process was carried out in 40 cycles, as follows: initial denaturation stage (95 °C, 5 min), followed by 10 cycles of denaturation stages (95 °C, 1 min), annealing (60 °C, 30 sec), extension stage (72, 1 min), then 30 cycles of denaturation (95 °C, 1 min), annealing (40 °C, 30 sec), extension stage (72 °C, 1 min), and final extension (72 °C, 10 min).

PKS-II gene amplification was carried out by mixing primer pairs PF6 (5′-TSGCSTGCTTGGAYGCSATC-3′) and PR6 (5′TGGAANCCGCCGAABCCGCT-3) (El Samak et al., 2018) with a concentration of 10 mM, 0.5 µL each, 0.5 µL of extracted DNA template, 7.5 µL of Thermo Scientific2X Phire Plant Direct PCR Master Mix, and 6 µL of ddH_2_O. PCR amplification was carried out in 30 cycles, as follows: initial denaturation (96 °C, 5 min), followed by denaturation (96 °C, 1 sec), annealing (58 °C, 1 min), extension (72 °C, 1.5 min), and the final extension (72 °C, 10 min).

#### 2.2.7. 16S rRNA amplification of active isolates

Amplification of the 16S rRNA gene was carried out by mixing 1 µL of DNA Template, primer pair 27F (5′-AGAGTTTGATCCTGGCTCAG-3′) and 1429R (5′-GGTTACCTTGTTACGACTT-3′) [16] with a concentration of 10 mM, 1 µL each, 12.5 µL of Thermo Scientific2X Phire Plant Direct PCR Master Mix, and 9.5 µL of ddH2O. PCR was carried out in 40 cycles with the following steps: initial denaturation (98 °C, 5 min), denaturation (98. 5 sec), annealing (55 °C, 5 sec), extension (72 °C, 1 min), and final extension (72 °C, 1 min).

#### 2.2.8. DNA visualization, sequencing and sequence analysis

Electrophoresis, the application of electric current to DNA samples with agarose gel media, is an important step in the DNA visualization process. 1% agarose gel, 1% agarose powder dissolved in TAE buffer solution (Tris Acetate EDTA) mixed with GelRed dye for UV light visualization, was used in this study. The electrophoresis process was carried out at a voltage of 100 volts, with a current of 400 A for 30 min. The agarose gel was then transferred to a UV Transilluminator for visualization of the DNA bands.

The amplified samples were sequenced to determine the sequence of nucleotide bases using the Sanger Deoxy Method. Sequencing results were edited using MEGA 7.0 software, then data from 16s rDNA primers were matched with data from GenBank NCBI.

**Table 1.** Bacterial morphology of antifungal strain candidates from Karimunjawa.

No	Isolate Code	Morphology	Elevation	Margin	Color
ISP 1 Medium					
1	KI 1-1	Round with scalloped margin	Flat	Undulate	White
2	KI 1-2	Round with scalloped margin	Convex	Undulate	Cream
3	KI 1-3	Irregular	Flat	Undulate	Yellow
4	KI 1-4	Round with raised margin	Crateriform	Undulate	Cream
5	KI 1-5	Round with raised margin	Crateriform	Undulate	Cream
6	KI 1-6	L-shaped	Raised	Undulate	White
7	KI 1-7	Irregular	Umbonate	Undulate	Brown
8	KI 2-1	Round	Flat	Entire	Brown
9	KI 2-2	Round	Raised	Entire	Milk white
10	KI 2-3	Round	Flat	Entire	Brown
11	KI 2-4	Round	Flat	Entire	Brown
12	KI 2-5	Round	Flat	Ciliate	Clear brown
13	KI 2-6	Concentric	Raised	Entire	Dark brown
14	KI 3-1	Round with raised margin	Crateriform	Ciliate	Brownish yellow

PYA Medium					

15	KP 1-1	Round	Convex	Ciliate	Clear white
16	KP 2-1	Thread-like	Flat	Entire	White

HVA Medium					

17	KH 1-1	Filamentous	Flat	Branching	White
18	KH 1-2	Round	Convex	Entire	Gray
19	KH 1-3	Concentric	Flat	Entire	Gray
20	KH 2-1	Round	Convex	Entire	Black
21	KH 2-2	Round	Raised	Entire	Black
22	KH 2-3	Round	Raised	Ciliate	Black
23	KH 2-4	Round	Umbonate	Undulate	Black
24	KH 2-5	Irregular	Raised	Undulate	White
25	KH 3-1	Concentric	Crateriform	Undulate	White
26	KH 3-2	Round	Umbonate	Ciliate	White
27	KH 3-3	Round	Raised	Undulate	Brown
28	KH 3-4	Round	Flat	Undulate	Black
29	KH 3-5	Round	Convex	Entire	Reflective brown
30	KH 3-6	Filamentous	Raised	Wooly	White
31	KH 3-7	Round	Convex	Entire	Yellow

#### 2.2.9. Mapping simulation of Biosynthetic Gene Cluster (BGC)

Biosynthetic Gene Cluster (BCG) mapping simulation was achieved by submitting the accession number of the whole genome sequence of the same bacterial species as the result of molecular identification of active isolates in AntiSMASH 6.0 (https://antismash.secondarymetabolites.org/).

**Table 2.** Bacterial morphology of antifungal strain candidates from Mangkang, Semarang.

No	Isolate Code	Morphology	Elevation	Margin	Color
PYA Medium					

1	SP 1-1	Round	Growing to the inside	Entire	Brown
2	SP 2-1	Round	Convex	Entire	Pink
3	SP 2-2	Punctiform	Flat	Undulate	Clear yellow
4	SP 2-3	Round	Flat	Undulate	White
5	SP 2-4	Round	Flat	Entire	Yellow
6	SP 2-5	Round	Flat	Entire	Brown
7	SP 3-1	Irregular	Flat	Undulate	White
8	SP 3-2	Round	Convex	Entire	Yellow
9	SP 3-3	Round	Raised	Wooly	Black and white
10	SP 3-4	Concentric	Raised	Entire	White
11	SP 3-5	Round	Flat	Entire	Black
12	SP 3-6	Irregular	Raised	Lobate	White
13	SP 3-7	Round	Raised	Wooly	White
14	SP 3-8	Round	Growing to the inside	Entire	Clear yellow
15	SP 3-9	Punctiform	Flat	Lobate	Light gray

HVA Medium					

16	SH 1-1	Round with radiating margin	Flat	Undulate	White and green
17	SH 1-2	Round with raised margin	Flat	Ciliate	White
18	SH 1-3	Irregular	Flat	Lobate	White and black
19	SH 1-4	Irregular	Flat	Undulate	White and black
20	SH 2-1	Round	Flat	Undulate	White
21	SH 3-1	Round	Flat	Entire	Brown
22	SH 3-2	Concentric	Flat	Ciliate	Brown
23	SH 3-3	Round	Flat	Ciliate	White
24	SH 3-4	Irregular	Raised	Undulate	Pink
25	SH 3-5	Concentric	Flat	Irregular	White
26	SH 3-6	Round with radiating margin	Raised	Undulate	White
27	SH 3-7	Concentric	Flat	Branching	White and gray
28	SH 3-8	Round	Raised	Entire	Gray

## 3. Results

Bacterial isolation from Karimunjawa mangrove sediments using three different media types resulted in 31 isolates, of which there were 14 isolates on ISP 1 media, 2 isolates growing on PYA media, and 15 isolates growing on HVA media as shown in Table 1. Meanwhile, bacterial isolation from sediment samples from Mangkang, Semarang, showed that there were 28 isolates, where no isolates were found growing on ISP 1 media, 15 isolates were found to grow on PYA media, and 13 isolates were found to grow on HVA media as shown in the Table 2.

**Figure 1.**
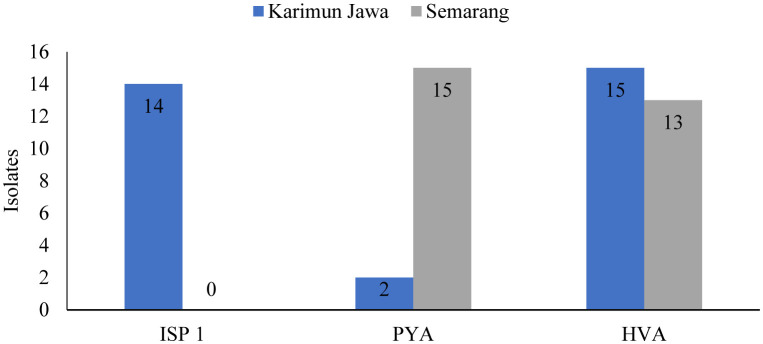
Bacterial isolates of antifungal strain candidates based on collection site and culture media.

The results of the isolation of bacterial samples showed different shapes, elevations, edges and colors of each isolate. The shape of the bacteria found were round, curved, irregular, rounded with raised margins, round and concentric, and filamentous. Of the 59 isolates collected from Karimunjawa and Semarang coastal areas, the dominant bacterial shape was circular (43%). The coloration of bacterial isolates found varies greatly from white, cream, yellow, red, brown, gray and black. Colonies with white coloration were found in most samples (20%).

**Table 3.** Antifungal activity data of bacterial isolates.

No	Isolate	Origin	Zone of Inhibition diameter against Pathogenic species (in cm)		
			*C. albicans*	*Trichoderma* sp.	*A. niger*
1	SP 2-4	Mangkang, Semarang	-	-	0.88 ± 0.11
2	SP 3-5		-	1 ± 0.04	1.57 ± 0.08
3	SP 3-8		0.58 ± 0.03	1.31 ± 0.07	0.80 ± 0.06
4	SP 3-9		0.33 ± 0.01	-	-
5	SH 3-4		-	1.08 ± 0.03	1.31 ± 0.06
6	KI 1-6	Karimun Jawa	-	-	1.53 ± 0.18
7	KI 2-2		-	-	0.82 ± 0.03
8	KI 2-4		-	-	1.26 ± 0.16

Based on the number of isolates of bacteria from the Karimunjawa and Semarang mangrove sediments cultured in three different media, PYA media produced the most isolates from the Semarang sample (15 isolates), HVA media produced the most isolates from the Karimunjawa sample (15 isolates), whereas ISP media produced 14 isolates from Karimunjawa samples without resulting any from the Semarang samples. These results are presented in Table 1.

**Figure 2.**
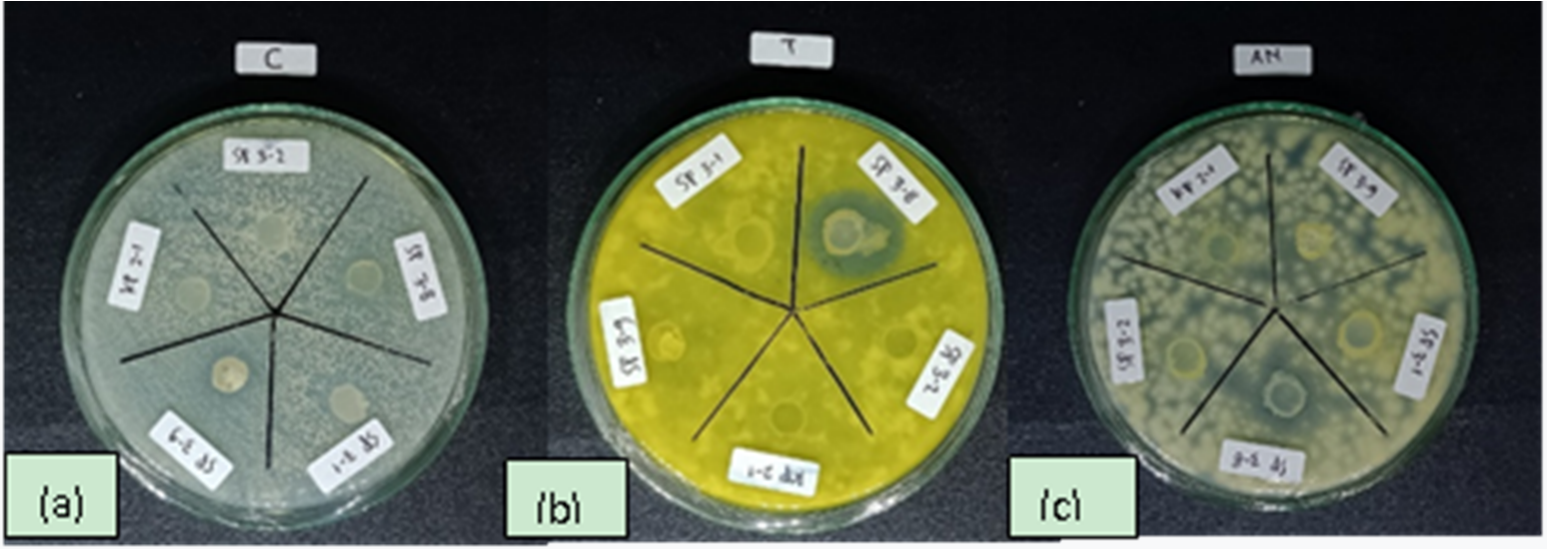
Isolate (SP 3-8) tested positive against *C. albicans* (0.58 ± 0.03 mm) (a), *Trichoderma* sp. (1.31 ± 0.07 mm) (b) and *A. niger* (0.80 ± 0.06) (c).

### 3.1. Antifungal activity

The screening of antifungal activities on bacterial isolates samples from all collection sites showed that there were 5 isolates from Semarang that were positive for the tested pathogenic species, namely isolates SP 2-4, SP 3-5, SP 3-8, SP 3-9 and SH 3-4. 3 isolates from Karimunjawa showed positive results against tested pathogenic species, namely Isolates KI 1-6, KI 2-2 and KI 2-4. Of all the positive results, only one isolate (SP 3-8) tested positive against *C. albicans* (0.58 ± 0.03), *Trichoderma* sp. (1.31 ± 0.07) (Figure 2) and *A. niger* (0.80 ± 0.06).

**Table 4.** Encoding genes for secondary metabolites.

No	Isolate	Origin	Encoding genes for secondary metabolites		
			NRPS	PKS	
				PKS I	PKS II
1	SP 2-4	Mangkang, Semarang	-	-	✓
2	SP 3-5		-	✓	-
3	SP 3-8		-	✓	✓
4	SP 3-9		✓	-	-
5	SH 3-4		✓	-	-
6	KI 1-6	Karimunjawa	✓	-	✓
7	KI 2-2		✓	✓	✓
8	KI 2-4		✓	-	✓

### 3.2. Detection of biosynthetic Gene Cluster (BGC)

Detection of Biosynthethic Gene Cluster (BGC) showed that the genes encoding secondary metabolites (NRPS, PKS 1 and PKS 2) were detected in KI 2-2 isolates from Karimunjawa. NRPS genes were detected only in isolates SP 3-9, SH 3-4 (From Mangkang, Semarang), KI 1-6, KI 2-2, KI 2-4 (From Karimunjawa islands). The secondary metabolite-encoding gene, PKS1, was detected in isolates SP 3-5, SP 3-8 (From Mangkang, Semarang), KI 2-2 (From Karimunjawa islands). Whereas PKS-II genes were detected in isolates SP 2-4, SP 3-8 (From Mangkang, Semarang), KI 1-6, KI 2-2, and KI 2-4 (From Karimunjawa islands).

Based on the number of isolate reactions in screening antifungal activity, 3 sample isolates were selected from Semarang for the next stage of research; isolates SP 2-4, SP 3-5 and SP 3-8.

**Table 5.** BLAST homology of isolates SP 2-4, SP 3-5 and SP 3-8.

No	Isolate	Relative similarity	Query Cover	Percent Identify	E value	Acc Number
1.	SP 2-4	*Z. amylolytica* strain HN-181 16S ribosomal RNA gene	100%	100%	0.0	DG423481.1
2.	SP 3-5	*P. aeruginosa* strain A-25 16S ribomosal RNA gene	99%	93.14%	0.0	MT573198.1
3.	SP 3-8	*P. aeruginosa* strain QK-2 16S ribosomal RNA gene	100%	100%	0.0	MH746105.1

The PCR and electrophoresis processes that were applied to bacterial isolates from the samples produced several DNA bands on the media for the test as shown in Table 5.

**Table 6.** BGC mapping simulation results the *Zhouia amylolytica* sample.

Region	Region Location (Nucleotides)	Type	Most Simillar Known Cluster	Similarity
Region 3.1	342857-363693	Terpene	Carotenoid	28%

*Note: Genetic mapping simulation of *Z. amylolytica* (NZ_FPAG01000003).

The high correlation value between isolates SP 3-5 with the sequences in the database (93.14%) indicated that this molecular identification (*P. aeruginosa* strain A-25 16S) should be a close match at the genus level. While isolates SP 2-4 (*Z. amylolytica* strain HN-181 16S) and isolate SP 3-8 (*P. aeruginosa* strain QK-2 16S) were exact matches with the sequences found in the database (100%).

**Table 7.** Mapping simulation results of the Biosynthetic Gene Cluster (BGC) of the *P. aeruginosa* sample

Region	Region Location (Nucleotides)	Type	Most Simillar Known Cluster	Similarity
Region 1	96108-138540	NRPS	L-2-amino-4-methoxy-trans-3-butenoic acid	100%

Mapping simulations of *Z. amylolytica* (NZ_FPAG01000003) and *P. aeruginosa* (KV830163.1) genes showed that *Z. amylolytica* genes contain carotenoids (28%), as shown in Table 6. It was also revealed that *P. aeruginosa* genes contains L-2-amino-4-methoxy-trans-3-butenoic acid, as shown in Table 7.

## 4. Discussion

Bacterial isolation was carried out from samples collected in sedimentary habitats in the mangrove ecosystem to obtain species that have antifungal potential. Different media were used in the isolation process. This method aims to maximize the number of bacterial isolates produced from the process. The results of bacterial isolation from mangrove sediments produced the most results in ISP 1 medium (14 isolates) from Karimunjawa sample, although the medium did not produce isolates from the Semarang sample. On the other hand, the PYA medium only resulted in 2 isolates from the Karimunjawa sample but managed to produce 15 isolates from the Semarang sample. HVA medium produced almost the same number of isolates between Karimunjawa samples (15 isolates) and mango samples (13 isolates). The difference in the number of isolates in Karimunjawa and Semarang with different media showed different results. A medium had an effect on the growth of isolates. A good growth medium is a medium that could provide a source of carbon and other minerals needed for growth and activities. Microorganisms need nutrients to support cell growth, the nutrients needed are carbon (C), nitrogen (N), phosphorus (P), sulfur (S), potassium (K), magnesium (Mg), calcium (Ca), sodium (Na) and iron (Fe), while the micronutrients needed are copper (Cu), manganese (Mn), zinc (Zn), nickel (Ni), molybdenum (Mo), and cobalt (Co) [17]. Minerals have an important role in enzyme reactions as cofactors in metabolic processes [18]. The combination of mineral mixtures also plays an important role in electrolyte and osmotic regulation in cells and small amounts. Minerals have an influence on cell growth and product formation. From the statements, it can be concluded that the minerals contained in the medium are also capable of supplying energy for the growth of isolates, so it is suspected that no isolates were found in the ISP 1 medium in Semarang because the media incidentally has not been able to supply isolate growth energy. The condition at the sampling point is abnormal condition because ISP 1 media should have a lot of isolates growing because ISP 1 is richer in nutrients [19].

Of the total 59 isolates produced from the sample isolation process, 31 isolates from Karimunjawa and 8 isolates from Semarang showed activity against test pathogenic bacteria, namely *C. albicans*, *Trichoderma* sp. and *A. niger*, which consisted of 3 Karimunjawa isolates and 5 Semarang isolates. Of the 8 isolates, there was only 1 isolate that was active against the three tested pathogenic bacteria; isolates SP 3-8.

Prior to identification of bacteria with potential as antifungals, NRPS, PKS 1 and PKS 2 were detected, namely to determine the presence or absence of PKS 1 or 2 genes. The results showed that the PKS samples SP 2-4, SP 3-5 and SP 3-8 meaning that the bacteria produced higher polyketide active compounds. Bacterial results by molecular method showed a 100% match with *P. aeruginosa* strain QK-2. While isolate SP 3-5 showed 93.14% similarity with *P. aeruginosa*. Based on the molecular identification of isolate SP 3-5, the isolate was believed to be close to the genus *Pseudomonas*. The results of the molecular identification of SP 2-4 proved that the isolate had 100% similarity with *Z. amylolytica*.

*Pseudomonas* sp. are commonly found in mangrove sediments and is known to possess antibacterial capability [20]. This species works in synergy with 3 other bacterial species, *Flavobacterium* sp., *Acinetobacter* sp., and *B. subtilis*, which collectively have the potential to combat the growth of pathogenic species, to the extent that they are proven to remove strong odor from waste. The consortium of bacteria also saw application in turning organic waste into compost [21].

The bacterium *P. aeruginosa* is known to be one of six pathogenic bacteria consisting of *Enterococcus faecium*, *Staphylococcus aureus*, *Klebsiella pneumoniae*, *Acinetobacter baumannii*, *P. aeruginosa*, and *Enterobacter* spp., which are generally associated with antimicrobial resistance, and also known by the abbreviation ESCAPE [22]. This information supports that *P. aeruginosa* is a dangerous bacterial pathogen, and that the species can survive antibiotics. This is presumably because this type of bacteria has toxic secondary metabolites that can kill fungi.

Based on gene mapping simulations, it is known that this species has a compound L-2-amino-4-methoxy-trans-3-butenoic acid. l-2-Amino-4-methoxy-trans-3-butenoic acid (AMB) is a potent antibiotic and toxin produced by *P. aeruginosa*. *P. aeruginosa* gene contains L-2-amino-4-methoxy-trans-3-butenoic acid as shown in Table 7. *P. aeruginosa* toxin L-2-amino-4-methoxy-trans-3-butenoic acid (AMB) is a non-proteinogenic amino acid which is toxic for prokaryotes and eukaryotes [23]. This means that the potential of these bacteria is known to be toxic to microorganisms and other biota. It is proven by the research results shown in the Table 7, namely as an anti-fungal.

The results of research conducted by Pringgenies et al. [24] found that the supernatant resulted from the extraction of *P. aeruginosa* found in symbiosis with the gastropod *Cerithidea* sp. contains Pyocyanin (pyo) and Phenazin I-carboxyic (PCA) pigments, which falls into the category of phenazine pigments. The results of studies on pyocyanin showed that when pyocyanin biosynthesis was inhibited, a decrease in the pathogenicity of *P. aeruginosa* was observed in vitro. This suggests that pyocyanin is most responsible for the initial colonization of *P. aeruginosa* in vivo [25].

The bacterial sample has a striking green color, which comes from the pigment phenazine.

Phenazine is a natural pigment which plays a vital role as anti-cancer, anti-malaria, anti-tumor and antibiotic agent. It is these characteristics that are believed to complicate the recovery of patients suffering from *P. aeruginosa* infestation-induced infections, as stated in. *P. aeruginosa* has the ability to form biofilm [26]. Antibiotic treatment was unable to eradicate bacterial infections with this biofilm-forming ability because of the intrinsic tolerance and development of resistance caused by mutations. Biofilm tolerance to antibiotics is multifactorial, involving physical, physiological, and genetic determinants.

On the other hand, bacterial antibiotic resistance in biofilms is caused by mutations and driven by repeated exposure of bacteria to high levels of antibiotics. This is interesting information because in pathogenic bacteria there are compounds that have the potential to be anti-cancer. The role of biotechnology in the form of fractionation will obtain promising results in the pharmaceutical field in the future.

The mapping simulation of the *Z. amylolytica* gene (NZ_FPAG01000003) revealed that this species possesses carotenoids (28%). Carotenoids are known to contain antioxidants that can protect cells from free radical damage. Several species are known to possess or produce carotenoids. The bacteria *Erythrobacter* sp. strain KJ5 which is a symbiote organism of the coral *A. nasuta*, is known to have zeaxanthin and -carotene [27]. In addition, it is also known that these bacteria do not have bacteriochlorophyll, but have at least 16 types of carotenoids, including -carotene and zeaxanthin [28]. Another research on compounds in *Erythrobacter* sp. strain KJ5 found that this species produces sulphur containing carotenoids, caloxanthin sulfate and nostoxanthin sulfate, in abundance [29].

*Z. amylolytica* is a species that thrives in sedimentary ecosystems, does not form spores, and was first isolated in samples collected from the South China Sea [30]. This species is known to be applied as a solution to the sediment problem of saltwater aquaculture ponds with Malachite green (MG) contamination [31]. This demonstrates the potential of *Z. amylolytica* as an antifungal, which has a wide range of applications in the aquaculture industry and other bioindustries.

## 5. Conclusions

Of the total 59 isolates produced from the sample isolation process, 31 isolates from Karimunjawa sediments and 8 isolates from Semarang sediments showed activity against test pathogenic bacteria, namely *C. albicans, Trichoderma* sp., and *A. niger*. Detection of Biosynthethic Gene Cluster (BGC) showed that the genes encoding secondary metabolites (NRPS, PKS 1 and PKS 2) were detected in KI 2-2 isolates from Karimunjawa. NRPS were detected only in isolates SP 3-9, SH 3-4, KI 1-6, KI 2-2, KI 2-4. The secondary metabolite-encoding gene, PKS1, was detected in isolates SP 3-5, SP 3-8, KI 2-2. PKS II genes were detected only on isolates SP 2-4, SH 3-8, KI 1-6, KI 2-2, and KI 2-4. Isolate SP 3-5 was revealed as *P. aeruginosa* (93.14%), isolate SP 2-4 was *Z. amylolytica* strain HN-181 (100%) and isolate SP 3-8 was *P. aeruginosa* strain QK-2 (100%).
